# Injectable platelet-rich plasma-based hydrogels: a controlled release platform for growth factors and cardiac regeneration post-myocardial infarction

**DOI:** 10.1093/rb/rbag082

**Published:** 2026-04-24

**Authors:** Yaqiang Shi, Yaqin Liu, Xiaoyu Bai, Wentao Gao, Yawei Ma, Jieneng Wang, Yuan Hu, Yongjie Jiang, Wei Li, Shifeng Zhou, Quan Qi

**Affiliations:** The First Clinical Medical College, Lanzhou University, Lanzhou 730030, China; Department of Cardiac Function Examination, Gansu Provincial Hospital of Traditional Chinese Medicine, Lanzhou 730050, China; The First Clinical Medical College, Lanzhou University, Lanzhou 730030, China; Department of Cardiovascular Surgery, The First Affiliated Hospital of Xi’an Jiaotong University, Xi’an 710000, China; Department of Cardiovascular Surgery, The First Hospital of Lanzhou University, Lanzhou 730030, China; Department of Cardiovascular Surgery, The First Hospital of Lanzhou University, Lanzhou 730030, China; Department of Cardiovascular Surgery, The First Hospital of Lanzhou University, Lanzhou 730030, China; The First Clinical Medical College, Lanzhou University, Lanzhou 730030, China; The First Clinical Medical College, Lanzhou University, Lanzhou 730030, China; The First Clinical Medical College, Lanzhou University, Lanzhou 730030, China; The First Clinical Medical College, Lanzhou University, Lanzhou 730030, China; Department of Cardiovascular Surgery, The First Hospital of Lanzhou University, Lanzhou 730030, China

**Keywords:** myocardial infarction, injectable hydrogel, platelet-rich plasma, myocardial regeneration, cardiac function

## Abstract

Myocardial infarction (MI) is a leading cause of high global cardiovascular mortality and disability. Platelet-rich plasma (PRP), as a widely available autologous biological product rich in various growth factors, holds significant potential for promoting tissue healing and regeneration. However, its clinical application is limited by short *in vivo* retention time and transient therapeutic effects, necessitating the development of novel drug delivery systems to enhance its clinical value. In this study, a novel injectable PRP-Gel composite was developed. Based on the Schiff base reaction, the composite gel exhibits excellent biocompatibility, biodegradability, appropriate mechanical properties and enables controlled release of multiple growth factors from PRP. Results at 28 days post-intramyocardial injection demonstrated that, compared with the control group, the PRP-Gel composite effectively promoted angiogenesis, inhibited cardiac cell apoptosis and injury, significantly reduced infarct size, decreased myocardial fibrosis and preserved ventricular wall thickness, ultimately leading to a marked improvement in cardiac function. In summary, the composite gel developed in this study provides an optimized strategy for the clinical application of PRP and represents a promising therapeutic approach for myocardial regeneration following MI.

## Graphical Abstract

**Graphical Abstract:**
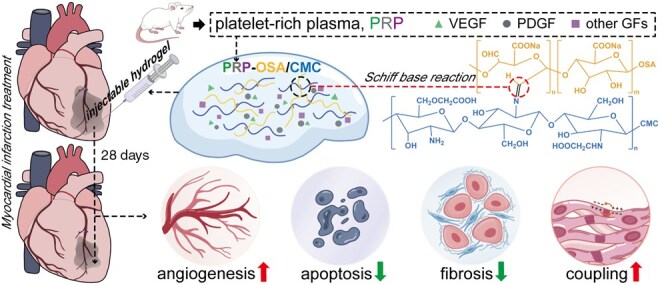

## Introduction

Myocardial infarction (MI) is a leading cause of cardiovascular mortality worldwide. The core challenge in its treatment lies in the irreversible loss of cardiomyocytes in the infarcted area, the subsequent adverse ventricular remodeling and the progressive decline in cardiac function [1, 2]. Although revascularization techniques such as percutaneous coronary intervention can rapidly restore blood flow, they cannot salvage necrotic myocardial tissue, and the incidence of post-procedural heart failure remains high [3]. Post-MI ventricular remodeling is a complex, multi-dimensional process involving structural, electrophysiological and autonomic nervous system changes. This includes cardiomyocyte hypertrophy, interstitial fibrosis, insufficient angiogenesis, sympathetic overactivation and distributional remodeling (e.g. abnormal increase in tyrosine hydroxylase-positive nerve fiber density) and electrophysiological remodeling (e.g. abnormal distribution of connexin 43) [4–6]. These factors collectively form the substrate for malignant ventricular arrhythmias and heart failure, significantly increasing the risk of sudden cardiac death [7].

In this context, platelet-rich plasma (PRP), as an autologous regenerative therapy, demonstrates unique potential with minimal side effects *in vivo* [8]. PRP is a platelet concentrate prepared from autologous whole blood via centrifugation. Its core biological property lies in the high-concentration network of growth factors (GFs) and cytokines released upon platelet activation. Platelet alpha-granules contain over 30 bioactive molecules, including platelet-derived growth factor (PDGF), vascular endothelial growth factor (VEGF), transforming growth factor-beta 1 (TGF-β1) and epidermal growth factor (EGF). These molecules synergistically regulate angiogenesis, suppress excessive inflammation and promote tissue healing by activating signaling pathways such as PI3K/AKT and MAPK [9, 10]. For instance, PDGF promotes the proliferation of fibroblasts and smooth muscle cells, while VEGF directly induces the migration of vascular endothelial cells and lumen formation; their synergistic action accelerates angiogenesis in ischemic tissue [9, 11]. Concurrently, TGF-β is crucial for regulating the balance between tissue generation and remodeling, influencing the complex processes involved in tissue homeostasis, and serves as a primary mediator of tissue regeneration and remodeling [12, 13]. Furthermore, PRP can activate the Piezo1 ion channel in cardiovascular cells, which is essential for cardiomyocyte regeneration [14].

Numerous studies have demonstrated that PRP exerts multifaceted cardiovascular protective and reparative effects through the release of various GFs [9, 15–17]. The underlying mechanisms include: promoting angiogenesis and myocardial repair; mitigating oxidative stress and stabilizing mitochondrial function to protect the myocardium from ischemia/reperfusion injury and improving cardiac systolic function, as evidenced by an increase in left ventricular ejection fraction (LVEF). Furthermore, PRP effectively inhibits adverse ventricular remodeling and reduces infarct size, thereby synergistically promoting cardiac recovery at both structural and functional levels. However, the bioactive constituents in free PRP are susceptible to rapid clearance *in vivo*, with a short half-life, which limits their capacity for sustained therapeutic efficacy [18]. Recently, Chen *et al.* [19] successfully prepared a novel PRP composite hydrogel loaded with Cu-TCPP-Mn nanosheets. This system effectively alleviates persistent inflammation in infected wounds and accelerates the healing process, providing important evidence for the carrier-based application of PRP. The aforementioned studies suggest that integrating PRP into biomaterial carriers can significantly amplify its therapeutic potential. Through precise regulation of the sustained release of bioactive constituents, this approach holds promise for more effective application in the treatment of MI.

In recent years, injectable hydrogels have provided revolutionary strategies for the precise repair of MI due to their minimally invasive nature, *in situ* gelation properties and potential for bioactive regulation [20–22]. These materials can form a gel *in situ* at the infarct site after minimally invasive injection, serving both as a mechanical scaffold to resist ventricular wall thinning and delay adverse remodeling, and as a sustained-release platform for therapeutic agents, enabling the on-demand controlled release of therapeutic factors [23–25]. Among numerous candidate materials, composite hydrogel systems based on oxidized sodium alginate (OSA) and carboxymethyl chitosan (CMC) exhibit distinct advantages due to their excellent raw material accessibility, biocompatibility, biodegradability and synergistic bioactivity [26]. The gel formation primarily relies on the Schiff base reaction between the aldehyde groups of OSA and the amino groups of CMC [27]. This system not only provides physical support but also possesses inherent cell affinity due to its polysaccharide backbone [28]. These physicochemical properties make OSA/CMC gels ideal scaffolds for myocardial tissue engineering.

However, while injectable hydrogels based on natural biomaterials possess desirable low toxicity, their functionality is limited and often insufficient to meet the complex biological signaling requirements for coordinated tissue regeneration [28]. The development of novel injectable biomaterials that combine biocompatibility with adequate functional properties is increasingly urgent for biomedical applications. Therefore, integrating the GF network of PRP with the controlled delivery capability of OSA/CMC hydrogels to construct a novel composite therapeutic system holds significant scientific value and application prospects. This study aims to optimize the gelation kinetics, mechanical properties and sustained-release behavior of bioactive factors in the composite gel by precisely regulating the oxidation degree (DO) and concentration of OSA, as well as its composite ratio with PRP. The comprehensive benefits and mechanisms of this PRP–OSA/CMC composite gel in inhibiting post-MI ventricular remodeling, promoting angiogenesis and reducing apoptosis will be systematically investigated. This work is intended to provide an innovative strategy for MI repair therapy that combines active biological regulation with appropriate mechanical support.

## Materials and methods

### Materials

Sodium alginate (SA, OKA, A58129), CMC (OKA, A53780), sodium periodate (NalO_4_, Aladdin, S104093) and hydroxylamine hydrochloride (SINOPHARM, 10011426). Rat VEGF ELISA kit (Jonln, JL21369), rat PDGF-BB ELISA kit (Elabscience, E-EL-R0537) and rat TGF-β1 ELISA kit (Jonln, JL12342). DMEM/HIGH GLUCOSE liquid medium (HyClone, SH30243.01B), premium fetal bovine serum (FBS, HyClone, SH30084.03) and penicillin–streptomycin solution (HyClone, SV30010). Cell Counting Kit-8 (CCK-8, Beyotime, C0037) and live/dead viability assay kit (Beyotime, C2015S). Bovine serum albumin (BSA, Sigma-Aldrich, B2064), DAPI (Roche, 10236276001), anti-alpha smooth muscle Actin (α-SMA) antibody (Mouse, Clone 1A4, Abcam, Cat# ab7817, 1:4,000 dilution), anti-CD31 antibody (Rabbit, Clone RM1006, Abcam, Cat# ab281583, 1:1,000 dilution), Beclin 1 Recombinant Rabbit Monoclonal Antibody (Clone JE59-31, HUABIO, Cat# HA721216, 1:600 dilution), LC3B Recombinant Rabbit Monoclonal Antibody (Clone JJ090-6, HUABIO, Cat# ET1701-65, 1:10000 dilution), Peroxidase AffiniPure Goat Anti-Rabbit IgG (H + L) (Polyclonal, Jackson ImmunoResearch, Cat# 111-035-003, RRID: AB_2313567, 1:400 dilution) and Peroxidase AffiniPure Goat Anti-Mouse IgG (H + L) (Polyclonal, Jackson ImmunoResearch, Cat# 115-035-003, RRID: AB_10015289, 1:200 dilution). TUNEL BrightRed Apoptosis Detection Kit (Vazyme, A113-01), 488 Tyramide Signal Amplification Kit (Runnerbio, Bry-880488) and 594 Tyramide Signal Amplification Kit (Runnerbio, Bry-880594). Detailed information regarding other materials is provided in Supplementary Table S1.

### Synthesis of OSA

OSA was synthesized by the selective oxidation of the cis-diol bonds in SA using NalO_4_ [29]. The procedure was briefly as follows: 5.0 g of SA was completely dissolved in 200 mL of deionized water. Under continuous stirring and nitrogen protection, specific amounts of solid NalO_4_ were added to the solution. The molar quantities of NalO_4_ corresponded to 20%, 40%, 60% and 80% of the molar amount of uronic acid units in SA, respectively, to prepare OSA samples with different theoretical degrees of oxidation. The reaction was carried out at 25°C for 24 h in the absence of light. After the reaction, 10 mL of ethylene glycol was added to quench the reaction and reduce the excess NalO_4_, with continuous stirring for 1 h. Subsequently, 4.0 g of sodium chloride (NaCl) and 800 mL of anhydrous ethanol were added to the mixture. The precipitate was collected, redissolved in deionized water and the mixture was dialyzed against deionized water using dialysis tubing with a molecular weight cutoff of 3000 for 5 days, with the water changed three times daily. Finally, the purified OSA solution was lyophilized to obtain OSA with different degrees of oxidation.

### Determination of the oxidation degree of OSA

The degree of oxidation of OSA was determined by the hydroxylamine hydrochloride method, with reference to an established procedure and minor modifications [30]. The procedure was briefly as follows: 0.05 g of dried OSA sample (denoted as *m*_OSA_) was accurately weighed and placed in a 150 mL conical flask. Then, 20 mL of 0.25 mol/L hydroxylamine hydrochloride solution (prepared in a 1:1 volume ratio of anhydrous ethanol to deionized water) was added, followed by 2–3 drops of bromophenol blue indicator. After stirring for 4 h, the mixture was titrated with a standardized 0.05 mol/L sodium hydroxide (NaOH) solution (denoted as C_NaOH_) until the color changed from yellow to blue-violet. The volume of NaOH consumed at the titration endpoint was recorded (denoted as V_sample_). A blank test without the OSA sample was conducted simultaneously, and the volume of NaOH consumed was recorded (denoted as V_blank_). The DO was calculated using the following formula:


(1) $$\mathrm{DO}\;(\%)=[C_{\mathrm{NaOH}}\times (V_{\mathrm{sample}}-V_{\mathrm{blank}})\times 198]/m_{\mathrm{OSA}}\times 100\%$$

where 198 (g/mol) represents the average molar mass of the repeating unit of SA.

### Preparation of PRP and quantification of GFs

Under sterile conditions, 8 mL of whole blood was collected from the carotid artery of Sprague-Dawley (SD) rats using a blood collection needle into a PRP serum separation tube (Sanli Medical Technology Development Co., Ltd., China). The tube was gently inverted 3–5 times and then allowed to stand upright for 15–20 min. The tube was centrifuged at 3000 rpm for 10 min using a centrifuge (DM0412, DLAB Scientific Co., Ltd., China). After centrifugation, the tube separated into three distinct layers: from top to bottom, these were platelet-poor plasma (PPP), PRP, separation gel and the cellular components. The upper PPP layer was slowly aspirated using a syringe, leaving behind 10–20% of the original blood collection volume, yielding PRP with a platelet concentration 4–6 times that of the rat whole blood (Supplementary Figure S1). The concentrations of VEGF, PDGF-BB and TGF-β1 in the freshly prepared PRP were quantitatively measured according to the manufacturer’s instructions of the respective rat ELISA kits. All measurements were performed using three independent PRP preparation samples, with each sample assayed in triplicate.

### Preparation of PRP–OSA/CMC composite hydrogel, optimization of gelation time and macroscopic characterization

Using phosphate buffered saline (PBS) as the solvent, a 6.5% (w/v) CMC pre-solution and a series of OSA pre-solutions with different degrees of oxidation (20%, 40%, 60%, 80%) and concentrations (10% and 20%, w/v) were prepared separately via ultrasonic oscillation at room temperature. Subsequently, a basic hydrogel formulation was identified through preliminary screening. Equal volumes of the CMC solution and the different OSA solutions were mixed, and the gelation time (defined as the time interval from solution mixing until flow cessation) was determined using the inverted vial method. A sample was considered to have formed a gel if no visible flow was observed within 30 s after vial inversion [31]. Based on the criteria of gelation time within a few minutes, good raw material solubility, and injectability, suitable combinations of OSA OD and concentration were selected as the base formulation for subsequent PRP composite hydrogel [32]. Subsequently, the preferred OSA/CMC basic solutions were mixed with different volumes of fresh PRP (specific ratios are provided in Supplementary Table S2). The inverted vial method was similarly employed to preliminarily screen formulations with gelation times meeting the requirements for injectability. Key gelation behaviors and macroscopic properties of the hydrogels were characterized. The gel-forming ability, injectability, self-healing property and viscoelasticity of the hydrogels were systematically evaluated using the inverted vial method, needle injection method, blade cutting method and finger flexion method, respectively.

### Rheological characterization of hydrogels

This study employed a ‘two-step screening method’ to optimize the composite hydrogel formulation. First, the vial inversion method was used for preliminary screening to identify formulations with gelation times suitable for the operational window of intramyocardial injection. Second, systematic rheological testing was performed to ultimately determine the optimal formulation. The methodology was referenced from relevant studies [33, 34]. The rheological properties of the hydrogel were comprehensively characterized using a Haake Mars III rotational rheometer (Thermo Scientific, Germany). Measurements were performed with a 25 mm parallel plate geometry, and the samples were trimmed to a thickness of 1 mm to ensure complete filling of the gap. All tests were conducted at 37°C to simulate physiological conditions, with three parallel samples tested per group. Initially, an amplitude sweep (strain range: 0.1–8000%) was performed at a fixed frequency of 1 Hz to determine the linear viscoelastic region (LVR, defined as the strain range where the storage modulus (*G*′) and loss modulus (*G*″) remain constant) and the critical strain point (the point where *G*′ intersects *G*″). Subsequently, a frequency sweep (frequency range: 0.1–100 Hz) was conducted within the determined LVR (at a selected strain of 1%) to evaluate the frequency dependence of the hydrogel and its solid-like behavior. Finally, the shear-thinning behavior and self-healing capability of the hydrogel were assessed via an alternating step strain test. This test comprised four cycles, each alternating between a low strain of 1% (maintained for 50 s, simulating a static state) and a high strain of 1000% (maintained for 50 s, simulating the high shear forces during injection). The recovery of the *G*′ was monitored to calculate the recovery ratio.

### Characterization of hydrogel microstructure

The morphological structure and pore size distribution of the crosslinked hydrogel network were examined using scanning electron microscopy (SEM). The hydrogels were rapidly frozen in a −80°C deep-freezer and subsequently transferred to a freeze-dryer for complete lyophilization. The dried samples were then fractured to expose their internal structure and mounted vertically onto the sample stage using conductive adhesive. After sputter coating with gold, the microstructure of the hydrogels was imaged using a scanning electron microscope (Quanta FEG 250, FEI, USA). Finally, the obtained SEM images were randomly sampled using ImageJ software (v.1.46r, The National Institutes of Health, USA). The diameters of at least 100 different pores were manually measured, and the average pore size was calculated.

### Characterization of compressive mechanical properties of hydrogels

The compressive mechanical properties of the hydrogels were characterized using a computer-controlled electronic universal testing machine (single column, 100 N capacity, Beijing Jitai Keyi, China). The test specimens were cylindrical in shape, with a diameter and height of 15 and 5 mm, respectively (*n* = 3 per group). Testing was performed at room temperature. A constant displacement rate of 2.5 mm/min was applied to subject the samples to axial compressive loading until structural failure occurred. The original load–displacement data acquired by the equipment in real-time were uniformly converted into engineering stress–strain curves. Based on these curves, key mechanical parameters were calculated and analyzed, including the Young’s modulus (determined as the slope of the initial linear region of the stress–strain curve, calculated via linear fitting), fracture strength (defined as the peak stress attainable in the stress–strain curve) and compressive toughness (characterized by calculating the integral area under the stress–strain curve from the origin to the point of fracture strain).

### Swelling property of hydrogels

Cylindrical hydrogels (1 mL) of equal volume were prepared from each group. After freeze-drying, the dry weight (W_d_) of each sample was accurately measured. The freeze-dried samples (*n* = 3) were then completely immersed in 5 mL of PBS. Under 37°C conditions, samples were retrieved at predetermined time intervals, gently blotted with filter paper to remove surface liquid, and immediately weighed (W_t_) until a constant mass was achieved (indicating swelling equilibrium). The swelling ratio was calculated using the following formula:


(2) $$\mathrm{Swelling}\;\mathrm{Ration}\;(\%)=(W_{t}-W_{d})/W_{d}\times 100\%$$

### Degradation property test

Under 37°C conditions, freeze-dried hydrogels (1 g) of equal mass from different groups were immersed in 5 mL of PBS (*n* = 3). At predetermined time points, samples were retrieved, gently rinsed with ultrapure water to remove degradation products and subsequently weighed (W*_t_*) after freeze-drying. The PBS solution was regularly replaced with fresh solution to maintain a stable degradation environment. The weight remaining percentage of the hydrogels was calculated as follows:


(3) $$\mathrm{Weight}\;\mathrm{remaining}\;(\%)=W_{t}/W_{d}\times 100\%$$

where W_d_ represents the initial mass of the freeze-dried hydrogel.

### *In vitro* release kinetics study

To evaluate the sustained-release performance of the PRP–OSA/CMC hydrogel for GFs, an *ex vivo* release test was conducted. A 100 μL aliquot of the hydrogel precursor solution (*n* = 3) was transferred into 1.5 mL centrifuge tubes and allowed to form a gel statically at 37°C. Subsequently, 1.0 mL of PBS release medium (pH 7.4) was added to each tube, which was then placed in a constant temperature oscillating incubator at 37°C and 120 rpm for incubation. At predetermined time points, the entire release medium was withdrawn and collected, followed by replenishment with an equal volume of fresh, pre-warmed PBS to maintain sink conditions. The collected samples were analyzed using corresponding ELISA kits to quantitatively determine the concentrations of VEGF and PDGF; all procedures were strictly performed according to the manufacturer’s instructions.

### *In vitro* hemolysis assay

The hemocompatibility of each experimental material was evaluated using a rat erythrocyte hemolysis assay. The procedure is briefly described as follows: whole blood was collected from healthy SD rats using sodium citrate as an anticoagulant. The blood was subsequently centrifuged at 3000 rpm for 15 min at 4°C to separate the erythrocytes, which were then washed three times with normal saline. The obtained erythrocytes were resuspended in normal saline to prepare a 4% (v/v) erythrocyte suspension. Equal volumes of the erythrocyte suspension were incubated with the positive control (double-distilled water), the negative control (normal saline) and the test samples (PRP, OSA/CMC hydrogel, PRP–OSA/CMC composite hydrogel) at 37°C for 2 h. Three parallel samples were set up for each group. After incubation, the mixtures were centrifuged at 3000 rpm for 15 min, and the supernatant was collected. The absorbance of the supernatant was measured at a wavelength of 540 nm using a microplate reader (MULTISKAN MK3, Thermo, USA). The hemolysis rate was calculated according to the following formula:


(4) $$\mathrm{Hemolysis}\;\mathrm{rate}\;(\%)=(D_{t}-D_{\mathrm{nc}})/(D_{\mathrm{pc}}-D_{\mathrm{nc}})\times 100\%$$

where D_t_, D_nc_ and D_pc_ represent the mean absorbance values of the test group, the negative control group and the positive control group, respectively.

### *In vitro* cytotoxicity evaluation

The *ex vivo* cytotoxicity of the hydrogels was evaluated using the CCK-8 assay and live/dead cell staining, with rat H9C2 cardiac cells as the model.

#### Preparation of extracts

OSA/CMC hydrogel and PRP–OSA/CMC composite hydrogel samples were immersed in DMEM complete medium containing 10% FBS at an extraction ratio of 0.2 g/mL. The mixtures were maintained statically in a 37°C incubator for 24 h for extraction. Subsequently, the extracts were collected and centrifuged at 3000 rpm for 15 min. The supernatants were sterilized by filtration through a 0.22 μm membrane filter to obtain 100% concentration stock extracts, which were stored at 4°C for later use. The stock extracts were diluted to concentrations of 25%, 50% and 75% using fresh complete medium.

#### Cell culture and treatment

H9C2 cells in the logarithmic growth phase were trypsinized, resuspended and seeded into 96-well plates at a density of 8000 cells per well (100 μL/well). The cells were pre-cultured for 24 h in a 37°C, 5% CO_2_ incubator. After cell adhesion, the original medium was aspirated and discarded. The experimental groups were treated with 100 μL of extracts at different concentrations (25%, 50%, 75%, 100%), respectively. The control group was replenished with an equal volume of fresh complete medium. To minimize the edge effect, the peripheral wells of the 96-well plate were filled with sterile PBS.

#### Determination of cell viability by CCK-8 assay

After exposure to the extracts for 1, 2 and 3 days, respectively, the liquid in the wells was aspirated and discarded. Then, 100 μL of fresh medium and 10 μL of CCK-8 solution were added to each well. Following an additional 2-h incubation period, the absorbance of each well was measured at a wavelength of 450 nm using a microplate reader. Cell viability was calculated according to the following formula:


(5) $$\text{Cell}\;\text{viability}\;\left(\%\right)=\left({\text{OD}}_{e}-{\;\text{OD}}_{b}\right)/\left({\text{OD}}_{c}-{\;\text{OD}}_{b}\right)\times 100\%$$

where OD_e_, OD_c_ and OD_b_ represent the absorbance values of the experimental group, control group (with cells) and blank group (without cells), respectively.

#### Live/dead cell staining

Furthermore, cells treated with the extracts for 24 h were stained using a Calcein-AM and Propidium Iodide double-staining kit. Hoechst 33342 live cell staining solution (Beyotime, C1028) was added at a 1:100 ratio. The cells were incubated at 37°C in the dark for 30 min. Subsequently, the morphology and distribution of live cells (green fluorescence) and dead cells (red fluorescence) were observed and recorded using an inverted fluorescence microscope (CKX53, Olympus).

### *In vitro* biological activity test

In the scratch assay, H9C2 cardiac cells (5 × 10^4^ cells/well) were seeded into 6-well plates. Upon reaching 90% confluence, a scratch was created using a 200 μL pipette tip. The cells were then incubated with serum-free medium containing PRP, OSA/CMC gel or PRP-Gel extract (with blank medium serving as the control). Images of the scratch were captured at 0 and 24 h, and the healing rate was calculated using ImageJ. For the Transwell assay, H9C2 cells (1 × 10^5^ cells/mL) were added to the upper chamber, while the lower chamber was filled with complete medium containing the aforementioned extracts. After 24 h of incubation, non-migrated cells were removed from the upper surface. The migrated cells were fixed, stained with crystal violet, and counted in five randomly selected fields. In the tube formation assay, Matrigel was solidified in 96-well plates. Human umbilical vein endothelial cells (HUVECs, 2 × 10^4^ cells/well) were then mixed with medium containing the extracts and seeded onto the gel (serum-free medium was used for the control group). After 8 h of incubation, the formation of vascular-like structures was observed, and the total tube length was quantified using ImageJ. All experiments were performed with three replicates per group and independently repeated three times.

### Establishment and treatment of the MI rat model

A rat model of MI was established using the coronary artery ligation method. The primary procedures were as follows: male SD rats (weighing 180–200 g) were anesthetized with sodium pentobarbital (30 mg/kg, intraperitoneal injection). Following tracheal intubation, mechanical ventilation was initiated using a VentStar small animal ventilator (Model R415, Shenzhen RWD Life Science Co., Ltd., China). A left thoracotomy was performed to expose the heart. The left anterior descending coronary artery was ligated using an 8–0 silk suture. Successful ligation was confirmed by significant ST-segment elevation on the electrocardiogram and pallor of the myocardium distal to the ligation site (Supplementary Video S1). Rats in the sham-operated group were subjected to identical surgical procedures except for coronary artery ligation. On day 7 post-MI, echocardiographic data were collected for baseline screening. Subsequently, the rats were randomly allocated into four groups: the PRP composite hydrogel group, the PRP-only group, the OSA/CMC hydrogel-only group and the saline control group. A second thoracotomy was then performed. The corresponding solutions were injected at four sites within the infarcted area (three sites in the border zone and one site in the infarct zone), with a total injection volume of 80 µL (Supplementary Video S2). All procedures were conducted under aseptic conditions.

All SD rats used in this study were purchased from Beijing Huafukang Bioscience Co., Ltd. The animals were housed in a specific pathogen-free (SPF) environment (temperature: 22 ± 2°C, humidity: 60–70%, 12 h/12 h light–dark cycle) with free access to food and water. The animal experimental protocol was approved by the Ethics Review Committee of the First Hospital of Lanzhou University (Approval No. LDYYLL2023-122) and strictly adhered to the Guide for the Care and Use of Laboratory Animals (NIH Publication No. 85-23, revised).

### Echocardiographic assessment

To dynamically evaluate cardiac structure and function, transthoracic echocardiography was performed on rats using a high-frequency small animal-specific ultrasound system (Vinno 6th Doppler Imaging System, VINNO6, Suzhou VINNO Technology Co., Ltd., China) on day 7 post-MI model establishment (pre-treatment baseline), and at 14 and 28 days post-treatment. The examination was conducted by an experienced sonographer. Clear 2D images and M-mode echocardiograms of the left ventricle were obtained from the parasternal long-axis view. The following parameters were analyzed and calculated using the workstation software: LVEF, left ventricular fractional shortening (LVFS), left ventricular internal diameter at end-diastole (LVIDd) and left ventricular internal diameter at end-systole (LVIDs). To minimize measurement error, all analyses were performed under blinded conditions (without knowledge of group allocation), and the average value from three consecutive cardiac cycles was taken as the final data for each parameter.

### Cardiac tissue harvesting and histological analysis

On postoperative day 28 following intramyocardial injection, rats were euthanized and tissues were harvested immediately. The hearts were perfused with 4% formaldehyde for 20 min and then rinsed in PBS for approximately 10 min. Subsequently, the entire heart was excised. From a point just below the ligature to the apex, the heart was serially sectioned transversely at 2 mm intervals, yielding five tissue blocks. All samples were fixed, routinely dehydrated, cleared, infiltrated with paraffin, embedded and sectioned coronally into 5-μm-thick serial sections. The section exhibiting the largest infarct area was selected for Masson’s trichrome staining to differentiate cardiac cells (red) from collagen fibers (blue). The stained sections were digitally scanned using a Pannoramic SCAN II whole-slide scanner (3DHISTECH Ltd., Budapest, Hungary) to obtain high-resolution panoramic digital images. Subsequent quantitative analysis of the digital slides was performed using ImageJ software. Specific parameters measured included: infarct zone wall thickness (mm) and the total fibrotic area (mm^2^) within the left ventricular (LV) wall. Each parameter was independently measured three times by two blinded observers, and the average value was taken as the final data. The myocardial fibrosis ratio was calculated as the percentage of the fibrotic area relative to the total area of the corresponding LV wall.

### Immunofluorescence and immunohistochemical staining

Paraffin-embedded heart tissue sections (5 μm thick) collected 28 days post-operation were utilized for staining analyses. All sections were pretreated by baking at 62°C, deparaffinization in xylene and rehydration through a graded ethanol series. Subsequently, antigen epitopes were retrieved using a heat-induced method (employing Tris-EDTA or EDTA buffer according to antibody requirements). Endogenous peroxidase activity and nonspecific binding sites were blocked with 3% H_2_O_2_ and 5% BSA, respectively.

Specific staining procedures were tailored to different targets. Immunohistochemical staining for CD68 was performed to evaluate macrophage infiltration. For CD31 and α-SMA double staining, a sequential tyramide signal amplification technique was applied. Sections were incubated sequentially with a mouse anti-α-SMA antibody (1:4000, Abcam, ab7817) and a rabbit anti-CD31 antibody (1:1000, Abcam, ab281583), followed by development with 594-tyramide and 488-tyramide reagents, respectively. Beclin-1 staining and LC3 staining were performed using rabbit recombinant monoclonal antibodies (Beclin-1: 1:600, HUABIO, HA721216; LC3B: 1:10 000, HUABIO, ET1701-65), respectively. For Connexin 43 (Cx43) and cardiac Troponin T (cTnT) double staining, sections were co-incubated with a mixture of the corresponding primary antibodies overnight at 4°C, followed by incubation with fluorescent secondary antibodies at room temperature. Apoptosis was detected using a TUNEL assay kit (Vazyme, A113-01) strictly according to the manufacturer’s instructions.

All stained sections were finally mounted with an anti-fade mounting medium containing DAPI or neutral balsam. Stained sections were imaged using a Pannoramic SCAN II digital slide scanner. Quantitative analysis was conducted within the infarct border zone. At least five randomly selected non-overlapping fields of view were analyzed independently by two observers blinded to the experimental groups. The following parameters were quantified: CD31^+^ vessel density, α-SMA^+^/CD31^+^ co-localization index (indicative of vessel maturity), the percentage of Beclin-1- and LC3-positive cells, the percentage of TUNEL^+^ apoptotic cells, the mean fluorescence intensity (MFI) of Cx43 and cTnT, and CD68^+^ cell density (cells/mm^2^).

### Statistical analysis

All data are presented as mean ± standard deviation (mean ± SD). Comparisons between two groups were performed using the independent *t*-test. For comparisons among three or more groups, one-way analysis of variance (ANOVA) was used under the assumption of homogeneity of variance. If significant differences among groups were detected, pairwise comparisons were further conducted using the Bonferroni *post hoc* test. For continuous variables (e.g. LVEF, LVIDd) measured at multiple time points (baseline, 14 days and 28 days post-myocardial injection), a two-way repeated measures ANOVA was employed to assess the main effects of time and treatment, as well as their interactions. Prior to analysis, Mauchly’s test of sphericity was performed; if *P* < 0.05, the Greenhouse–Geisser correction was applied. If a significant interaction was identified, simple effect analysis was conducted, with Bonferroni correction applied for multiple comparisons. All statistical analyses were performed using SPSS software (Version 24.0; IBM, USA). All tests were two-sided, and a *P*-value <0.05 was considered statistically significant.

## Results and discussion

### Preparation and primary characterization of hydrogels

**Figure 1:**
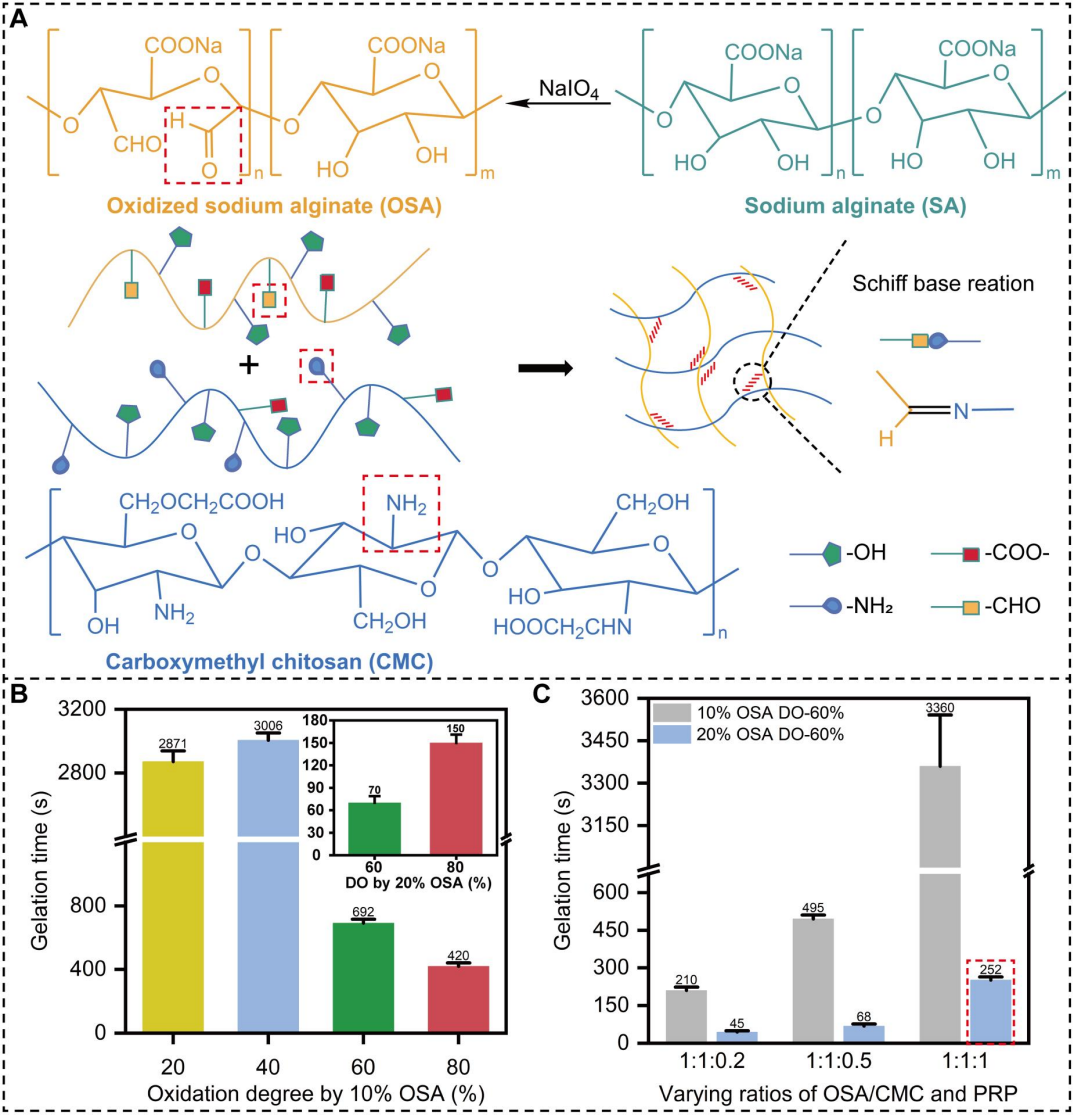
Construction and gelation time determination of hydrogels. (**A**) Schematic illustration of the cross-linking between OSA and CMC via Schiff base reaction. (**B**) Gelation time of OSA with different OD and concentrations (10% or 20% w/v) mixed with a 6.5% (w/v) CMC solution. (**C**) Effect of different PRP incorporation ratios on the gelation time of the composite gel (based on 60% oxidized OSA). Data are presented as mean ± standard deviation (*n* = 3).

**Figure 2:**
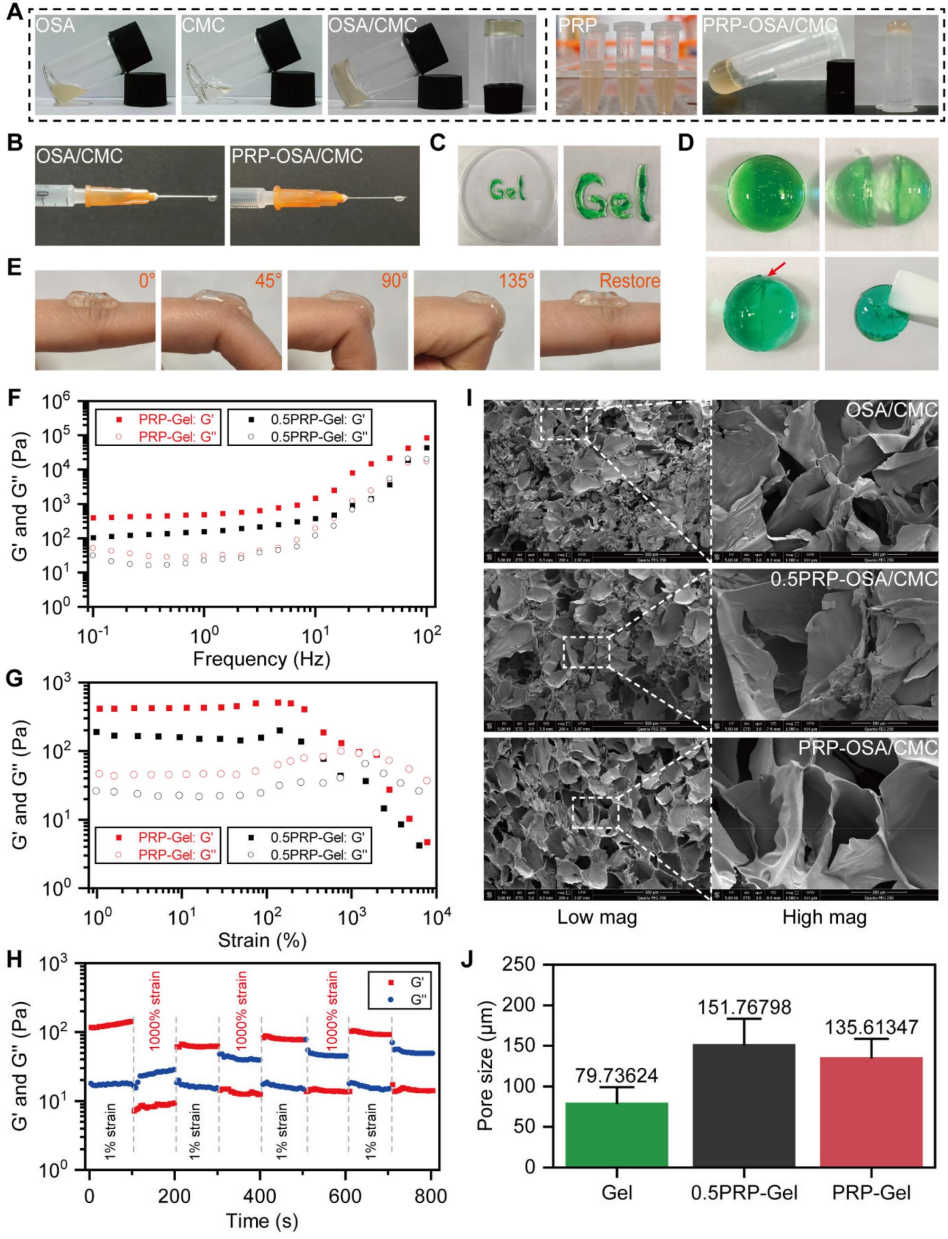
Macroscopic characterization, rheological properties and microstructural analysis of the hydrogels. (**A**) Macroscopic synthesis of OSA/CMC and PRP–OSA/CMC hydrogels. (**B, C**) injectability test. (**D**) Self-healing properties. (**E**) Viscoelastic test. (**F–H**) The rheological properties of the PRP composite hydrogels were evaluated through amplitude sweep, frequency sweep and alternating step strain sweep tests, respectively. (**I**) SEM morphology (scale bars: low magnification 500 μm, high magnification 100 μm). (**J**) Pore size distribution of the hydrogels. Data are presented as mean ± standard deviation (*n* = 3).

Injectable PRP composite hydrogel was fabricated based on the Schiff base reaction between OSA and CMC (Figure 1A). First, the concentration and DO of OSA were optimized through gradient screening. Subsequently, the incorporation ratio of PRP was adjusted, and further screening was performed using the vial inversion method and rheological analysis to ultimately determine the optimal formulation for the PRP composite gel. The results indicated that the addition ratio of PRP exerted a significant and concentration-dependent regulatory effect on the gelation behavior. As shown in Figure 1B and C, when the PRP ratio was low (OSA:CMC:PRP = 1:1:0.2), the gelation time was shortened to approximately 45 s. However, this excessively short gelation time was unfavorable for injection procedures and offered limited loading capacity for bioactive components; therefore, this ratio was not adopted. Furthermore, although OSA with a high DO (80%) achieved a suitable gelation time, its poor raw material solubility and injectability limited its application. Based on preliminary screening results, this study established a 20% (w/v) OSA solution with a 60% DO and a 6.5% (w/v) CMC solution as the foundational cross-linking system. Two representative PRP composite hydrogel formulations with gelation times suitable for the operational window of intramyocardial injection were selected for subsequent validation (volume ratios of 1:1:0.5 and 1:1:1, with gelation times of 68 and 252 s, respectively). Macroscopic characterization revealed that the hydrogels exhibited a light brownish-yellow color and could be smoothly extruded through a syringe, demonstrating excellent injectability (Figure 2A–C). After being cut, the fractured surfaces of the gel were observed to fuze upon contact for 30 min, and the healed hydrogel could withstand moderate stretching without rupture, indicating favorable self-healing properties (Figure 2D). Furthermore, the hydrogel could be bent to various angles on a finger and subsequently recovered its original shape, reflecting good flexibility and viscoelasticity (Figure 2E).

Further analysis of the rheological properties of the two composite gels after initial screening was conducted. Amplitude sweep results (Figure 2F) showed that the *G*′ of the composite gel with a 1:1:1 ratio (PRP-Gel) was approximately 450 Pa, falling within the range of 400–1800 Pa, which is close to the *G*′ value of native myocardial tissue. This characteristic is beneficial for constructing well-structured engineered myocardial tissue and is expected to reduce ventricular wall stress and mitigate myocardial remodeling [35, 36]. In contrast, the *G*′ of the 1:1:0.5 ratio gel (0.5PRP-Gel) was only about 180 Pa, significantly lower than that of myocardial tissue, and it exhibited a narrower LVR, indicating weaker resistance to deformation. Furthermore, the critical strain value of PRP-Gel was approximately 1000%, higher than that of 0.5PRP-Gel. When the strain exceeded this threshold, *G*′ sharply decreased and fell below the *G*″, indicating disruption of the gel network structure and a transition of the material from a solid to a liquid state [37]. Chen *et al.* [28] prepared a dual-crosslinked CMC–OSA–DTP hydrogel by incorporating 3,3′-dithiodipropionohydrazide (DTP), which demonstrated superior mechanical properties compared to the single-crosslinked OSA/CMC gel, with a critical strain of 240%. In comparison, PRP-Gel exhibited a higher critical strain value, suggesting enhanced tolerance to large deformations, which is advantageous for adapting to the complex strain environment of periodic cardiac motion. Frequency sweep tests further confirmed hydrogel formation and the stability of the gel network [38]. Within the frequency range of 0.1–100 Hz, the *G*′ of PRP-Gel consistently remained higher than its *G*″, and both moduli remained stable within the 0.1–10 Hz range (corresponding to physiologically relevant frequencies, such as heart rate) (Figure 2G). In contrast, 0.5PRP-Gel exhibited significant shear stiffening at high frequencies, potentially rendering it unable to withstand the periodic mechanical deformation of the heart. Additionally, alternating step strain sweep tests further demonstrated the remarkable self-healing capability of PRP-Gel. After undergoing four strain cycles, both moduli recovered to their initial values (Figure 2H). Furthermore, this gel exhibited typical shear-thinning behavior. Under high-strain conditions (1000%), *G*″ exceeded *G*′, and the material displayed fluid-like characteristics, ensuring smooth injection through fine-gauge needles. Once the high-strain condition was removed, the moduli rapidly recovered, indicating its suitability for intramyocardial injection in therapeutic applications. In summary, the composite gel with a 1:1:1 ratio (PRP-Gel) was ultimately selected for subsequent experiments in this study.

This suggests that PRP may function as a ‘dynamic modulator’ within this composite hydrogel system: at low concentrations, its Ca^2+^ ion cross-linking and pH buffering effects predominated, accelerating gelation; at high concentrations, physical dilution of the base components, competitive consumption of aldehyde groups by proteins and the shielding effect of high ionic strength collectively led to a prolonged gelation time [39, 40]. Through precise regulation, a composite hydrogel system optimized for injectability, gelation kinetics and functional loading was successfully obtained.

The microstructure of the hydrogels was characterized by SEM. As shown in Figure 2I and J, both the pure OSA/CMC hydrogel and the PRP composite hydrogel exhibited a 3D porous network structure. The network of the pure OSA/CMC hydrogel was densely crosslinked. After the introduction of an equal volume of PRP, the cross-linking density of the composite hydrogel network decreased, but the pore structure distribution became more uniform. Quantitative analysis indicated that the average pore size of the PRP-Gel was 135.6 ± 23.0 μm. This scale is conducive to the migration and infiltration of host cells while potentially maintaining necessary mechanical integrity [41, 42].

### Other characterization of the hydrogels

**Figure 3:**
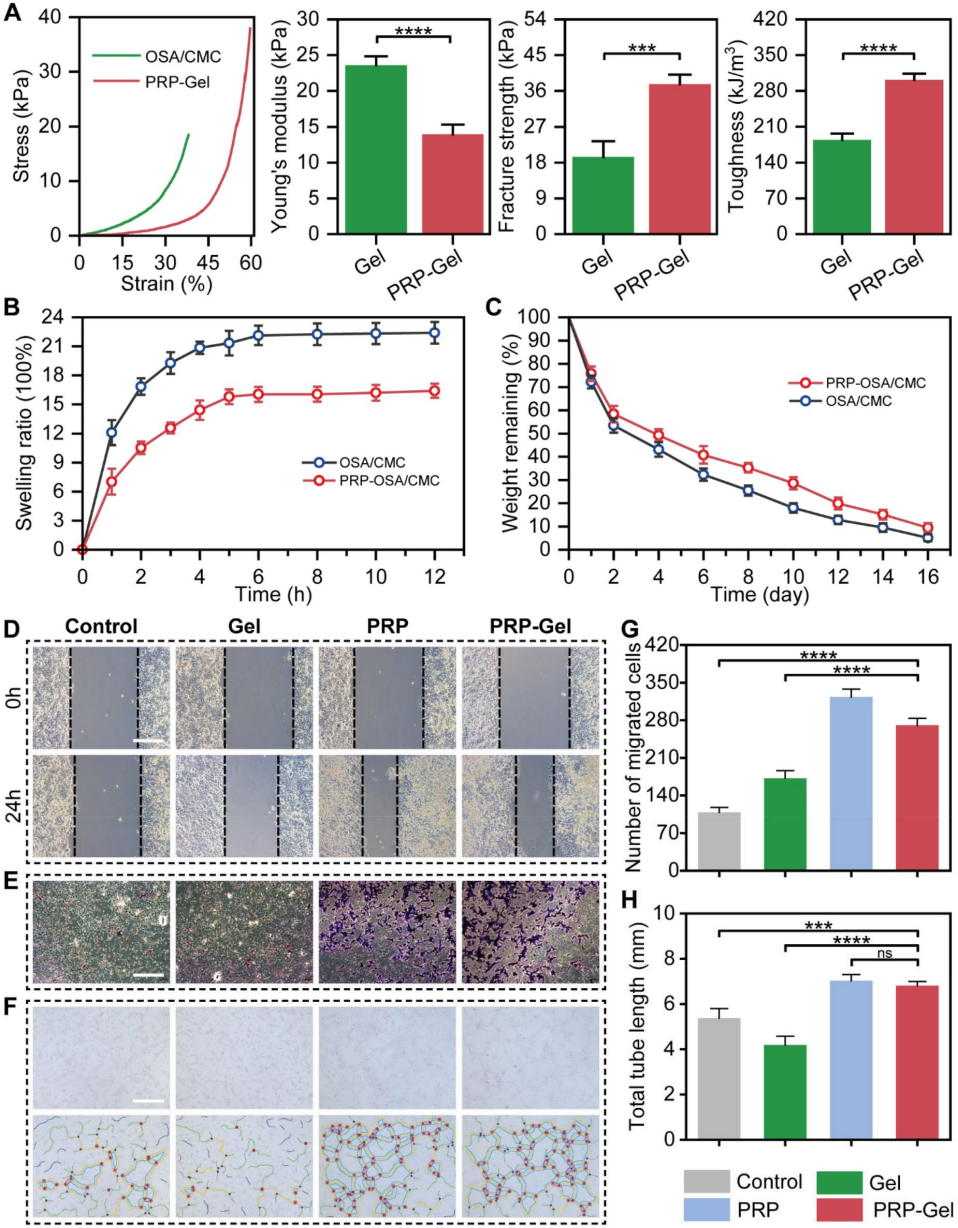
Other characterizations and *in vitro* biological activity assessment. (**A**) Compressive stress–strain curves, Young’s modulus, fracture strength and toughness of the hydrogels. (**B**) Swelling curve of the hydrogels. (**C**) Degradation curve of the hydrogels. (**D, E**) Cell scratch assay and Transwell assay of H9C2 cells (scale bar: 200 μm). (**F**) Tube formation assay of HUVEC (scale bar: 500 μm). (**G**) Number of migrated cells. (**H**) Total tube length. Data are presented as mean ± standard deviation (*n* = 3); ns indicates no significant difference, ***P < 0.001, ****P < 0.0001.

The mechanical properties of the hydrogels were characterized via compression mechanical testing (Figure 3A). The stress–strain curves revealed that the PRP-Gel could withstand approximately 60% compressive strain, demonstrating favorable deformation capability [43]. The Young’s modulus test results indicated that the incorporation of PRP reduced the Young’s modulus of the gel (14.0 ± 1.3 kPa) compared to the pure OSA/CMC gel. However, this appropriately reduced stiffness of the PRP-Gel better matched the physiological mechanical demands of cardiac cells [44]. This characteristic can minimize mechanical mismatch between the implant and myocardial tissue, avoiding stress shielding or stress concentration, which is beneficial for the functional integration of cells and thereby promotes cell adhesion and proliferation [45, 46]. Simultaneously, the PRP-Gel exhibited significantly enhanced fracture strength and compressive toughness. This suggests that during the continuous, periodic diastolic and systolic activities of the heart following MI, this material can not only provide suitable initial mechanical support, but its improved structural toughness and resistance to failure can better adapt to the dynamic and complex mechanical microenvironment. It is anticipated to maintain structural integrity and functional stability in the infarcted area over the long term.

To evaluate the *in vivo* applicability of the hydrogel, its swelling and degradation behaviors were systematically assessed in this study. The PRP-Gel demonstrated faster swelling equilibrium (approximately 5 h) and a lower equilibrium swelling ratio (approximately 16% at 12 h), which was significantly lower than that of the pure OSA/CMC hydrogel (Figure 3B). This indicates that the PRP-Gel could reduce the likelihood of generating additional mechanical stress on the surrounding myocardial tissue due to excessive swelling after implantation. Degradation performance tests revealed that the initial degradation rates of the two hydrogels were similar, but the difference gradually increased after 4 days. By day 16, the PRP composite hydrogel exhibited a higher residual mass (Figure 3C). Combined with the analysis of microscopic morphology and mechanical properties, the introduction of PRP, while maintaining a suitable pore size and matching the modulus of myocardial tissue, likely enhanced the stability of the cross-linked network through multiple interactions between proteins and polysaccharide chains (e.g. hydrophobic interactions, hydrogen bonds) [47]. This conferred superior structural retention and anti-degradation properties to the composite hydrogel, providing crucial assurance for its long-term mechanical support in the dynamic cardiac environment.

The GFs abundantly present in PRP, such as VEGF and PDGF, play a significant role in angiogenesis and myocardial repair [48–50]. However, in their free state, these factors are prone to rapid clearance, which limits their effectiveness in tissue engineering applications [51]. To evaluate the function of PRP composite gel as a controlled-release carrier for GFs, its release kinetics were analyzed in this study. As determined by ELISA quantification, the initial concentrations of VEGF, PDGF and TGF-β in fresh PRP under resting state were 12.0 ± 0.5, 50.5 ± 4.0 and 199.1 ± 10.4 pg/mL, respectively (Supplementary Figure S2). As shown in Supplementary Figure S3, representative GFs (VEGF and PDGF) in the composite gel exhibited a sustained-release trend during the initial 5 days, after which the release rate plateaued. By day 14, the cumulative release amounts of VEGF and PDGF were 27.8 ± 1.2 and 59.9 ± 2.0 pg/mL, respectively. This release profile indicates that the gel network can effectively load and retard the diffusion of GFs, which may be attributed to the dynamic cross-linking between the amino groups in PRP and the aldehyde groups in OSA, as well as the physical barrier effect of the gel network [19]. This mechanism helps maintain a locally safe and effective concentration while preventing rapid loss, thereby potentially exerting a more prolonged biological regulatory role in the myocardial microenvironment.

### *In vitro* biological activity assessment

The results of the cell scratch assay showed that the 24-h scratch healing rate in the PRP group and the PRP-Gel group was approximately 30% higher than that in the control group. In the Transwell assay, the number of migrated cells was significantly increased in both the groups. The tube formation assay demonstrated that the total length of HUVEC vascular-like structures was significantly greater in both the groups compared to the control group (Figure 3D–H, Supplementary Figure S4). These results confirm that PRP and its composite hydrogel can effectively promote cardiac cells migration and angiogenesis.

### *In vitro* biocompatibility analysis

**Figure 4:**
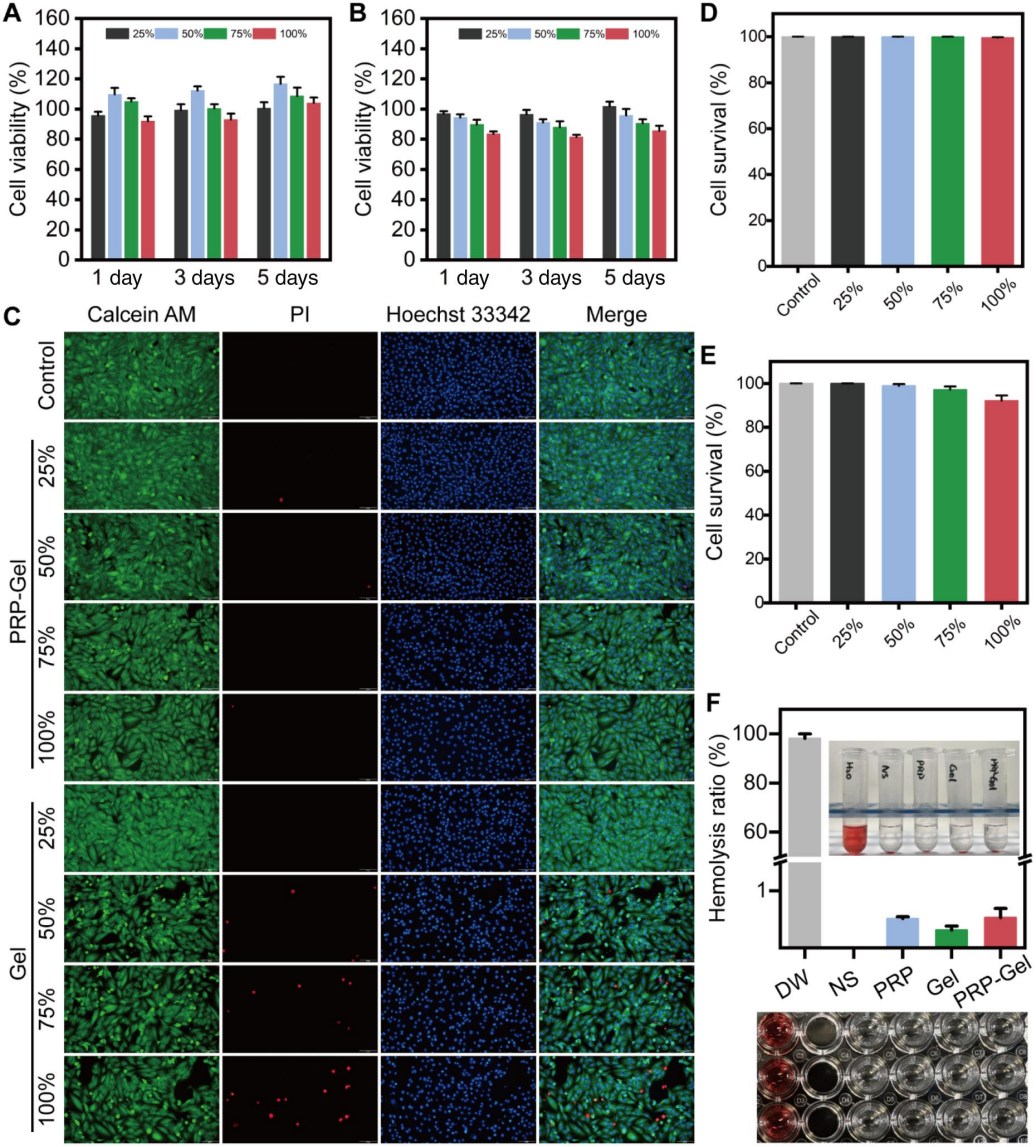
*In vitro* biosafety evaluation of the hydrogels. CCK-8 cytotoxicity assay of H9C2 cells treated with different concentrations of (**A**) the PRP composite hydrogel and (**B**) the OSA/CMC hydrogel. (**C**) Live/dead staining images of H9C2 cells after 24 h of treatment with extracts from different hydrogel groups (scale bar: 100 μm). Cell viability of H9C2 cells after direct co-culture for 24 h with different concentrations of (**D**) the PRP composite hydrogel or (**E**) the OSA/CMC hydrogel. (**F**) *In vitro* hemolysis rate of the hydrogel materials. Data are presented as mean ± standard deviation (*n* = 3).

A systematic assessment of the hydrogel’s *in vitro* biocompatibility was conducted in this study. The results of the CCK-8 assay (Figure 4A and B) indicated that the PRP composite gel exhibited favorable cytocompatibility. After co-culture with H9C2 cardiac cells, cell viability was significantly higher than that in the pure OSA/CMC gel group. Particularly, under the condition of 50% extract medium on day 5 of culture, viability reached 116.8 ± 4.6%, suggesting a positive role of PRP in promoting cell proliferation. Cell viability under all tested conditions exceeded 80%. According to the ISO 10993-5 standard, the cytotoxicity grade of the material was rated as 0, meeting the safety requirements for biomedical materials [52]. The results of live/dead cell staining further confirmed the good compatibility of the materials (Figure 4C–E). After 24 h of culture, the cell survival rate was above 99.6% in all PRP composite gel groups and above 92.2% in the OSA/CMC gel group, with very few dead cells observed, indicating that the material extracts did not cause significant cellular damage.

Furthermore, hemolysis assay results (Figure 4F) showed that the hemolysis rates of all hydrogel samples after co-incubation with rat erythrocytes were below the 5% safety threshold, and the supernatant was clear, demonstrating that the materials did not disrupt erythrocyte membrane integrity [53]. Biocompatibility is crucial for the functional performance of materials in clinical applications [52]. Further injection of the hydrogels into the myocardium of normal rats was performed, followed by HE staining of the cardiac tissue and major organs 3 days later. The results demonstrated a reversible, mild injection‑related reaction in the cardiac tissue. No significant inflammatory infiltration or pathological alterations were observed in the other major organs (Supplementary Figure S5). In summary, this PRP composite gel demonstrated good biosafety and biocompatibility, showing potential for further development as a medical material for myocardial repair.

### Injectable PRP-Gel composite improves cardiac function after MI

**Figure 5:**
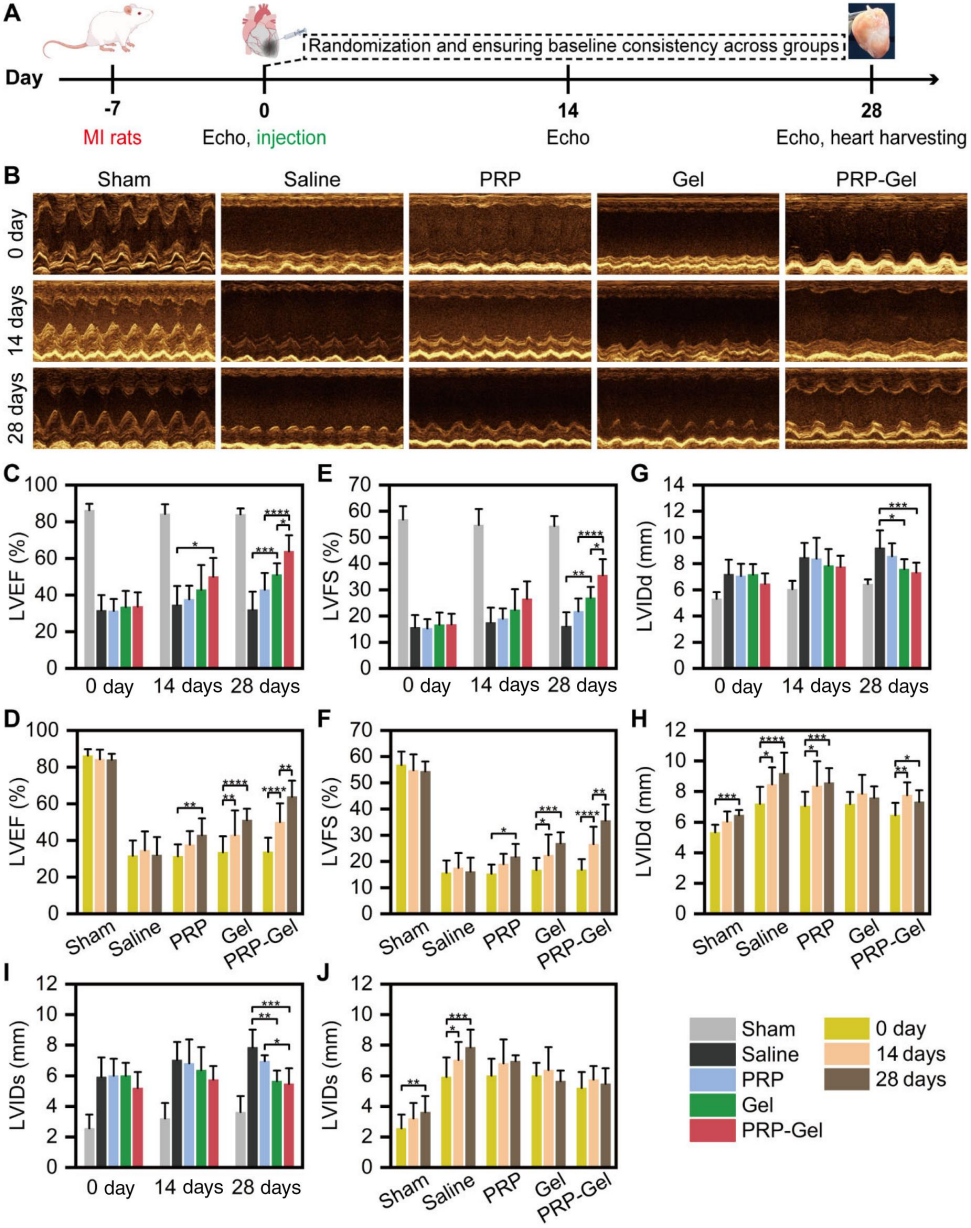
Echocardiographic evaluation of rats with MI following intramyocardial injection therapy. (**A**) Schematic diagram of the experimental protocol, including model establishment, therapeutic intervention, echocardiographic assessment and tissue harvesting. (**B**) Representative echocardiographic images from each group at different time points. (**C–J**) Analysis results of cardiac function parameters (two-way repeated measures ANOVA). Data are presented as mean ± standard deviation (*n* = 7); *P < 0.05, **P < 0.01, ***P < 0.001, ****P < 0.0001. Some elements in panel a were sourced from Figdraw (www.figdraw.com).

To evaluate the therapeutic efficacy of the PRP composite hydrogel, echocardiography was performed on rat models at day 7 post-MI (treatment baseline), as well as at 14 and 28 days after treatment (Figure 5A and B). A two-way repeated measures ANOVA was employed to assess the effects of time, treatment modality and their interaction on cardiac function parameters, including LVEF, LVFS, LVIDd and LVIDs.

The results revealed a significant interaction between time and treatment modality for all cardiac function parameters (*P* < 0.001), indicating that the pattern of cardiac function change over time differed depending on the intervention (Supplementary Table S3). Further simple effect analysis (Figure 5C–J) demonstrated that, compared to the saline control group, treatment with the PRP composite gel significantly improved LVEF and LVFS at both 14 and 28 days post-treatment. The most pronounced functional improvement was observed in this group by day 28. Although the PRP injection-only group showed improvement compared to its own baseline, no statistically significant difference was found versus the control group. This confirmed that, in the absence of a delivery vehicle, the bioactive constituents in PRP could not exert effective therapeutic action due to rapid enzymatic degradation and clearance *in vivo*. Regarding the inhibition of adverse ventricular remodeling (Figure 5G–J), both the hydrogel groups (OSA/CMC and PRP composite gel) significantly attenuated the post-infarction LV dilation process, as evidenced by significantly lower increases in LVIDd and LVIDs compared to the control group. However, it is noteworthy that in the OSA/CMC gel group, the post-treatment LVIDd showed no significant change from baseline. Its relatively high Young’s modulus and poor toughness may have limited the ventricle’s adaptive enlargement during diastole to some extent, potentially affecting physiological contractile reserve. In contrast, the mechanical properties of the PRP composite gel were more closely matched to those of native myocardium and it possessed superior toughness (Figure 3A). This allowed it to provide moderate constraint to prevent ventricular aneurysm formation while simultaneously permitting ventricular contraction and relaxation closer to the physiological state.

In summary, the PRP composite gel functioned through a dual mechanism combining mechanical compatibility and controlled release of bioactive factors: its optimized mechanical properties effectively restricted pathological ventricular dilation, while its function as a GF reservoir continuously modulated the local microenvironment. This synergistic action led to significant promotion of cardiac systolic function recovery at multiple time points, offering a material-based strategy with translational potential for MI therapy.

### Inhibition of ventricular remodeling and myocardial fibrosis

**Figure 6:**
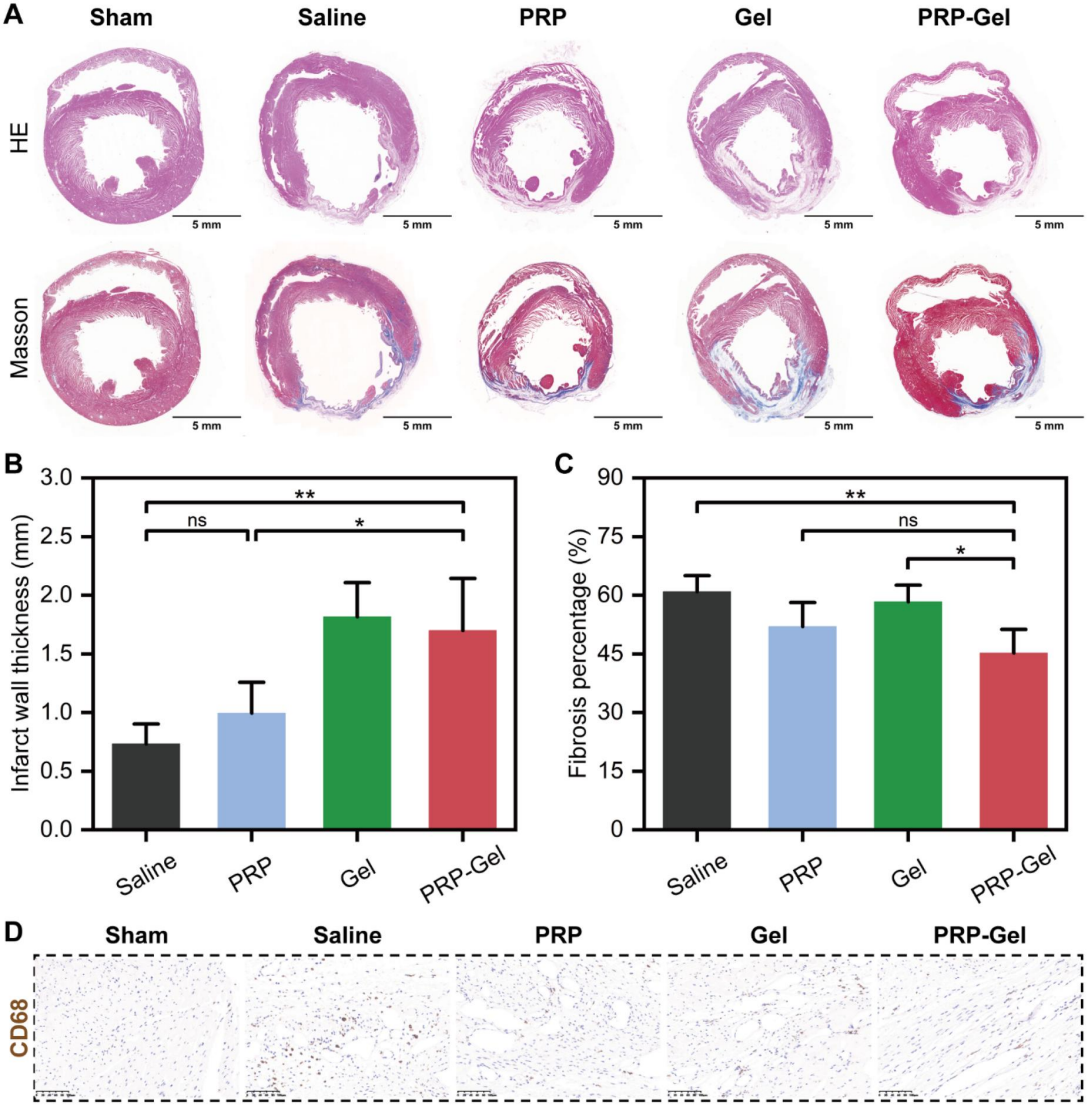
Masson’s trichrome staining, fibrosis analysis and CD68 immunohistochemical staining of myocardial tissue. (**A**) Representative HE staining and Masson’s trichrome staining images of myocardial tissue from each group after 28 days of treatment. (**B, C**) Quantitative analysis of infarct zone wall thickness and the percentage of collagen fiber area (based on Masson’s staining). (**D**) Representative images of CD68 immunohistochemical staining in the infarct border zone at 28 days post-treatment (scale bar: 100 μm). Data are presented as mean ± standard deviation (*n* = 5); ns indicates no significant difference, *P < 0.05, **P < 0.01.

Histological analysis at 4 weeks post-operation further confirmed the therapeutic efficacy of the PRP composite gel. Masson’s trichrome staining results (Figure 6A) demonstrated that hydrogel injection exerted a profound influence on the morphological remodeling of the post-infarct heart. Analysis of ventricular wall thickness (Figure 6B) indicated that, compared to the non-gel injection groups (normal saline: 0.7 ± 0.2 mm; PRP alone: 1.0 ± 0.3 mm), injection of gels into the infarct zone provided structural physical support to the damaged myocardium, significantly increasing the infarct wall thickness (OSA/CMC gel: 1.8 ± 0.3 mm; PRP composite gel: 1.7 ± 0.4 mm). More importantly, the PRP composite gel significantly reduced the myocardial fibrosis ratio (45.2 ± 6.0%) compared to the saline group (60.9 ± 4.1%) and the OSA/CMC gel group (58.3 ± 4.3%) (Figure 6C). It is noteworthy that under stimulation by inflammatory signals and GFs such as TGF-β, cardiac fibroblasts can differentiate into myofibroblasts, which exhibit enhanced proliferation and collagen synthesis capacity, representing a key mechanism of post-MI fibrosis [54]. PRP contains TGF-β, and its excessive release carries a risk of promoting fibrosis [55]. Although the sustained-release properties of the composite hydrogel and its design without exogenous activators were intended to ensure its safe and controllable release, its specific risks in MI treatment require further validation. Furthermore, PRP may potentially trigger an immune response in xenogeneic/allogeneic applications [56]. However, a significant reduction in CD68^+^ macrophage infiltration was observed at 28 days post-injection in this study (Figure 6D, Supplementary Figure S6), suggesting that the PRP composite hydrogel did not induce a harmful persistent immune response and may even help mitigate post-infarction inflammation, which could be one of the mechanisms underlying its inhibition of myocardial fibrosis.

These findings suggest that the PRP composite gel not only maintains ventricular structure through mechanical support but also inhibits myocardial fibrosis via the sustained release of bioactive signals, thereby enhancing the quality of cardiac repair [24]. This dual intervention in cardiac remodeling at both macroscopic and microscopic levels renders it an optimized therapeutic strategy capable of improving the prognosis of MI both structurally and functionally.

### PRP composite gel promotes angiogenesis and maturation

**Figure 7:**
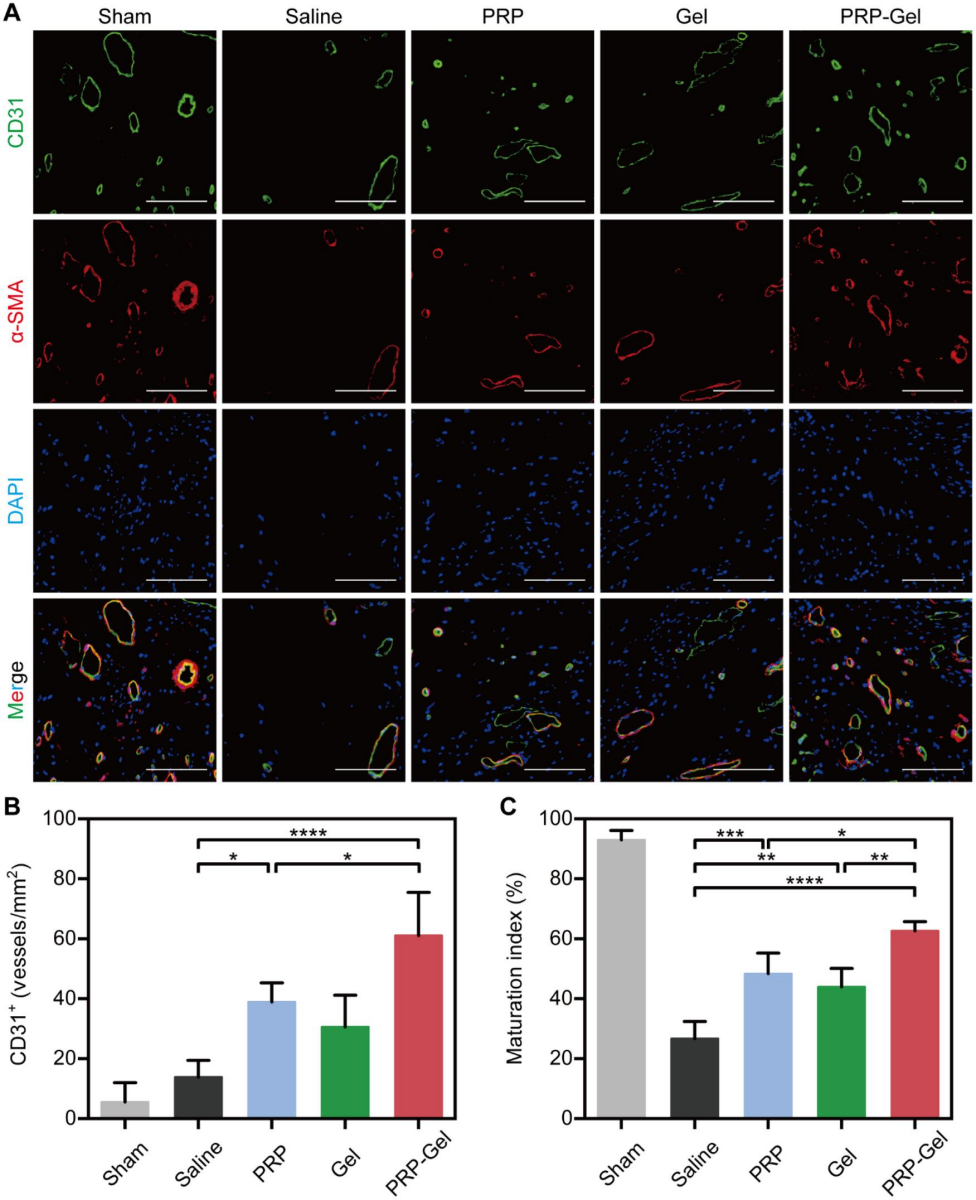
Assessment of angiogenesis and maturity in the MI border zone. (**A**) Representative immunofluorescence staining images of vessels in the infarct border zone at 28 days post-treatment: CD31 and α-SMA were used to label endothelial cells and smooth muscle cells, respectively; DAPI (blue) was used to label nuclei (scale bar: 100 μm). (**B**) CD31^+^ vessel density (vessels/mm^2^). (**C**) α-SMA vascular coverage (maturity index). Data are presented as mean ± standard deviation (*n* = 5); *P < 0.05, **P < 0.01, ***P < 0.001, ****P < 0.0001.

Angiogenesis is crucial for the survival and repair of infarcted myocardium [57]. Immunofluorescence staining for CD31-positive neovessels was performed in the peri-infarct region on day 28 post-injection (Figure 7A). Quantitative analysis revealed (Figure 7B) that the PRP composite gel group exhibited the most potent pro-angiogenic capacity (61.1 ± 14.3 vessels/mm^2^) compared to the other groups. The neovessel density in the PRP injection group (38.9 ± 6.4 vessels/mm^2^) was also significantly higher than that in the saline group (13.9 ± 5.6 vessels/mm^2^). As an autologous biological material, PRP contains various bioactive molecules that are essential for tissue survival and neovascularization within the myocardium [58]. This study further confirms the pro-angiogenic activity of GFs in PRP and highlights the critical role of the hydrogel carrier in maintaining a locally effective concentration and achieving sustained release. Further co-localization analysis of CD31 and α-SMA (Figure 7C) demonstrated that the vascular maturation index in the PRP composite gel group was also significantly higher than in all other groups. This indicates that the composite system not only induces neovessel formation but also promotes mural cell coverage and functional maturation, thereby establishing a more stable and effective perfusion network. This process contributes to enhanced necrotic tissue clearance, shortened inflammatory phase and reduced hypoxia-induced apoptosis, which serves as an important structural foundation for inhibiting fibrosis and improving cardiac function [9]. Western blot analysis revealed that on day 28 post-treatment, the ratios of p-Akt/Akt, p-ERK/ERK and p-eNOS/eNOS in the myocardial tissue of the infarct border zone were significantly upregulated in the PRP-Gel group (Supplementary Figure S7). These ratios were markedly higher than those in the PRP and Gel groups. These findings confirm that the composite hydrogel can synergistically activate the Akt/ERK/eNOS signaling pathway, elucidating its potential mechanism for promoting angiogenesis and improving the myocardial microenvironment at the molecular level.

### Effects of the PRP gel composite on apoptosis and autophagy

**Figure 8:**
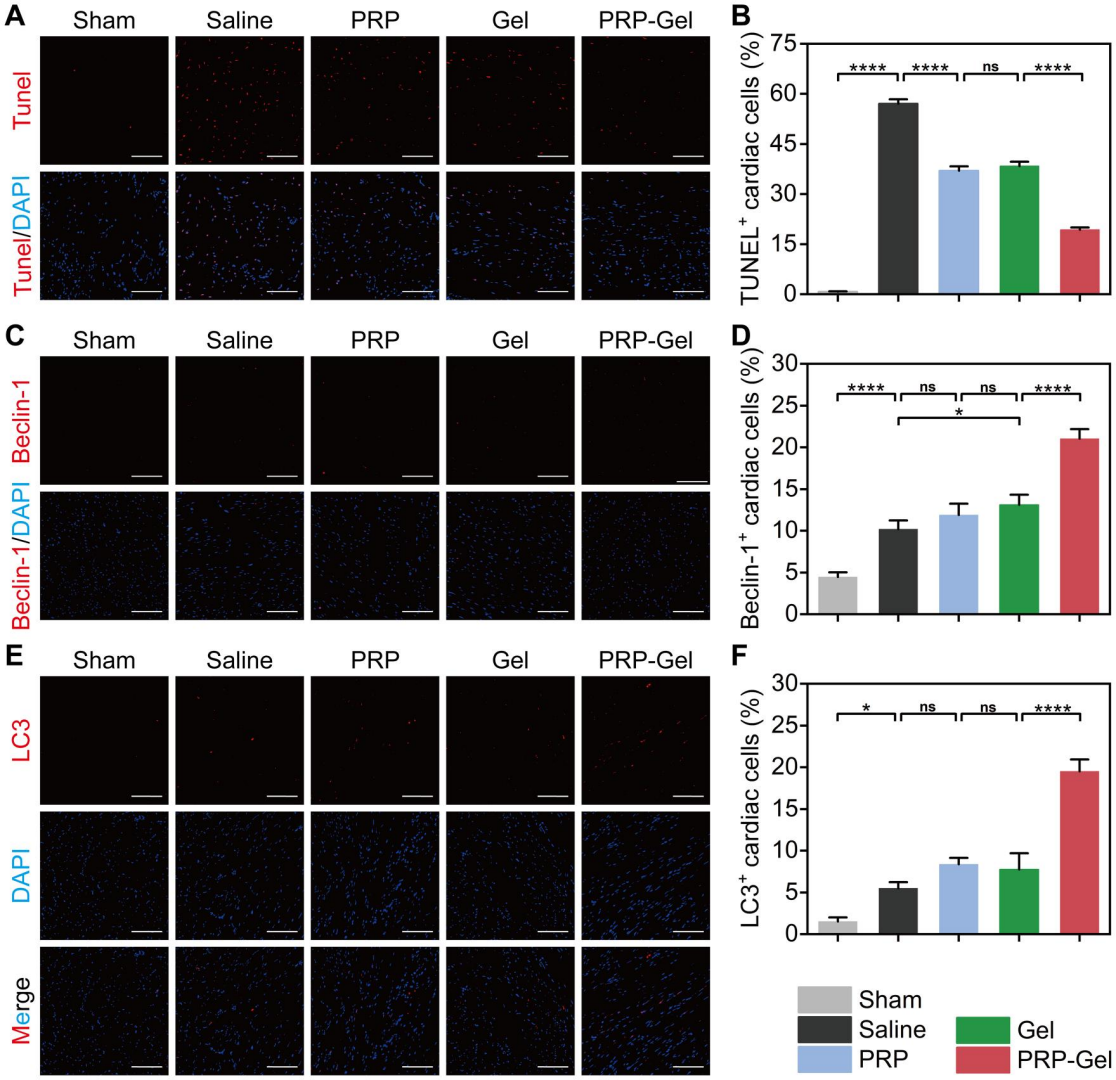
Evaluation of cardiomyocyte apoptosis and autophagy-related proteins. (**A**) Representative immunofluorescence images of TUNEL (apoptosis) and DAPI (nuclei) staining in the peri-infarct border zone at 28 days post-treatment (scale bar: 100 μm). (**B**) Percentage of TUNEL^+^ cells. (**C**) Representative immunofluorescence images of Beclin-1 staining in the peri-infarct region at the same time point (scale bar: 100 μm). (**D**) Beclin-1 positive rate. (**E**) Representative immunofluorescence images of LC3 staining (scale bar: 100 μm). (**F**) Percentage of LC3-positive cells. Data are presented as mean ± standard deviation (*n* = 5); ns indicates no significant difference, *P < 0.05, ****P < 0.0001.

Myocardial cell apoptosis is another primary cause leading to heart failure. Once myocardial cell loss occurs in the infarcted area, the surviving cardiac cells in the non-infarcted zone are subjected to increased pressure and/or volume overload, resulting in compensatory hypertrophy [59]. This compensatory response may transition to decompensation over the long term, leading to the death of residual myocardial cells in the peri-infarct tissue [60]. Apoptosis of myocardial cells in the peri-infarct area was assessed using the TUNEL staining method (Figure 8A), and the percentage of TUNEL-positive nuclei was quantitatively analyzed. The results (Figure 8B) demonstrated that the proportion of apoptotic cells in the PRP composite gel treatment group was significantly lower than that in the other groups (19.3 ± 0.7%). This indicates that this material can effectively inhibit the programmed death of myocardial cells, contributing to the preservation of both the quantity and function of viable cardiac cells in the peri-infarct region.

Recent studies have shown that the autophagic mechanism is significantly upregulated approximately 1–3 days after MI in rats, functioning to degrade and recycle damaged proteins and organelles, thereby supporting cellular metabolism. Subsequently, the autophagic flux process becomes impaired around 1 week post-infarction, failing to effectively support cell survival, which consequently induces necroptosis [59, 61]. In this study, the expression of the key autophagy regulatory protein Beclin-1 and the important marker LC3B was detected [62, 63], and the autophagic status of cardiomyocytes in the infarct border zone at 4 weeks post-injection was preliminarily assessed (Figure 8C and E). Quantitative analysis (Figure 8D and F) revealed that the positive rates for Beclin-1 and LC3 in the PRP composite hydrogel group were significantly higher than those in the saline control group (21.0 ± 1.2% vs. 10.2 ± 1.1% and 19.5 ± 1.4% vs. 5.5 ± 0.7%, respectively; both *P* < 0.0001). These two indicators collectively suggest the induction of the autophagic pathway. However, these findings reflect autophagosome formation rather than autophagic flux. Whether the observed autophagic activation represents enhanced autophagic flux or impaired autophagosome clearance requires further investigation using biochemical methods to elucidate. The aforementioned finding suggests that during the subacute phase of MI, the composite hydrogel may exert cardioprotective effects by moderately activating autophagy, promoting the clearance of damaged organelles and metabolic adaptation, increasing intracellular energy supply and thereby providing sufficient energy for cell survival [64, 65].

### Effects on cardiac cell integrity and Cx43 expression

**Figure 9:**
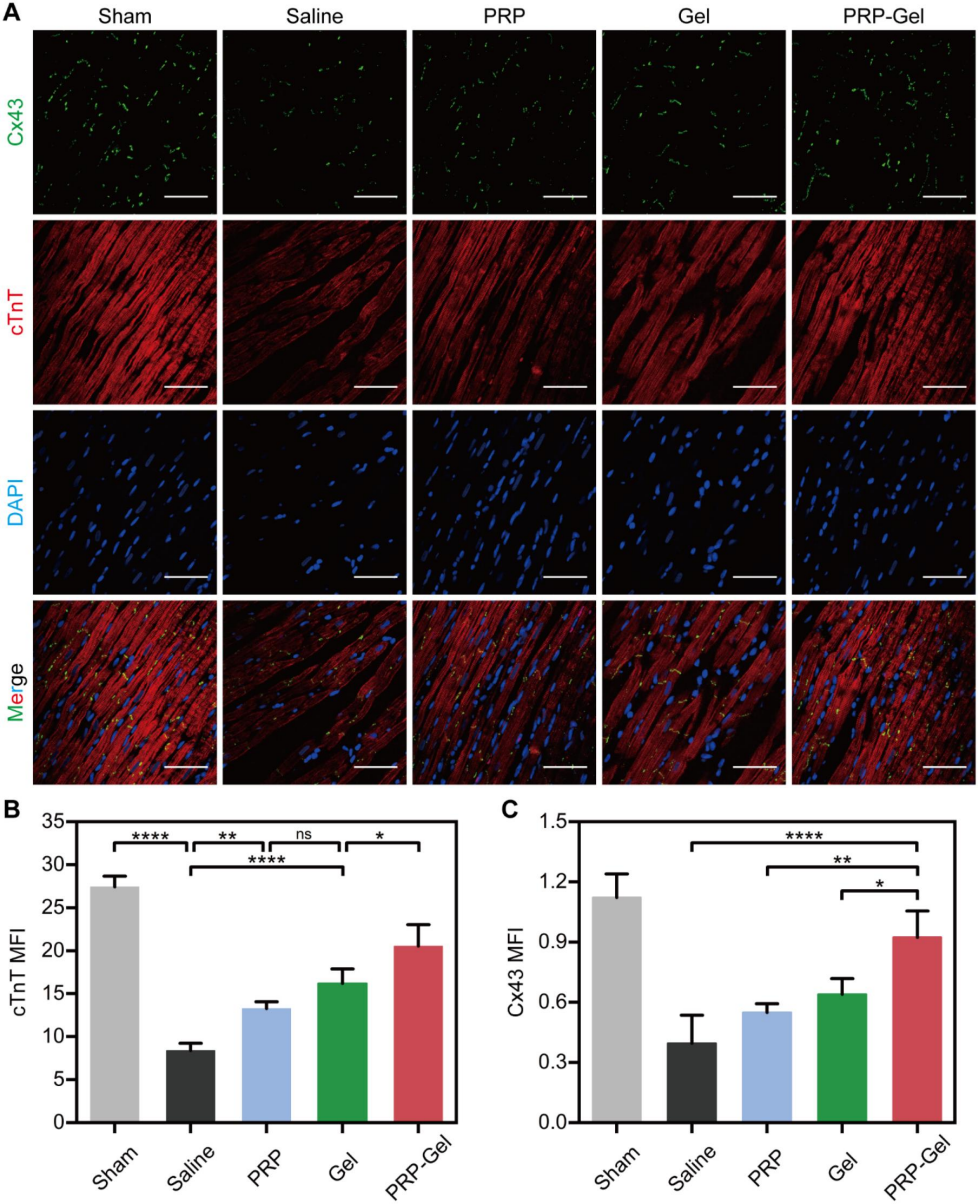
Expression of structural proteins and connexins in cardiac cells of the peri-infarct region. (**A**) Representative immunofluorescence staining images of cTnT and Cx43 in the peri-infarct region at 28 days post-treatment (scale bar: 50 μm). (**B**) Relative fluorescence intensity of cTnT. (**C**) Relative fluorescence intensity of Cx43. Data are presented as mean ± standard deviation (*n* = 5); ns, not significant; *P < 0.05, **P < 0.01, ****P < 0.0001.

To evaluate the impact of the PRP composite hydrogel on the structural integrity of cardiomyocytes and the structural basis related to electrophysiology, immunofluorescence staining for Cx43 and cTnT was performed on post-operative cardiac tissues (Figure 9A). The results (Figure 9B) demonstrated that, compared to the saline control group, treatment with the PRP composite hydrogel significantly increased the expression intensity of Cx43 in the peri-infarct zone (0.9 ± 0.1 vs. 0.4 ± 0.1, *P* < 0.0001). Following MI, the limited regenerative capacity of cardiac cells leads to the emergence of electrically silent areas within the infarct region, contributing to LV electrophysiological and structural remodeling [66]. Cx43 is the core protein constituting cardiac gap junctions [67, 68]. The upregulation of Cx43 expression and improved distribution observed in this study suggest that it may provide a favorable structural basis for electrochemical coupling between cells in the peri-infarct zone. This is expected to improve electrical conduction and potentially create conditions for reducing the risk of arrhythmias [69]. It is important to emphasize that direct electrophysiological functional validation was not conducted in this study. Therefore, the observed change in Cx43 expression can only reflect potential improvements at the structural level, and its impact on the restoration of functional electrical coupling requires confirmation in subsequent investigations. Concurrently, the cTnT fluorescence signal within the myocardial tissue of the PRP composite hydrogel group was also significantly enhanced (20.5 ± 2.5 vs. 8.4 ± 0.9, Figure 9C). While cTnT is a recognized biomarker of myocardial injury, the enhanced cTnT signal in the tissue does not directly equate to ongoing injury [70]. Instead, it more likely reflects a better-preserved density of viable and functional cardiac cells in that region. This finding corroborates the aforementioned observations of reduced apoptosis and enhanced angiogenesis, collectively indicating that the PRP composite hydrogel protects cardiac cells from loss through multiple pathways, thereby maintaining the number of contractile units.

## Conclusion

A novel injectable composite hydrogel based on OSA, CMC and PRP was successfully developed in this study. By optimizing the formulation ratio, this material system achieved mechanical properties matching those of myocardial tissue and an appropriate *in situ* gelation time, while also serving as a platform for the sustained release of GFs. Through the dual synergistic mechanisms of mechanical support and bioactive factor release, the PRP–OSA/CMC composite hydrogel effectively increased the ventricular wall thickness in the infarcted area, promoted functional angiogenesis and vascular maturation and inhibited cardiac cell apoptosis. Consequently, it synergistically ameliorated post-MI cardiac structure and function across multiple dimensions, offering a new material strategy with translational potential for myocardial repair.

## Supplementary Material

rbag082_Supplementary_Data
